# Co-exposure to multiple vitamins and the risk of all-cause mortality in patients with diabetes

**DOI:** 10.3389/fendo.2023.1254133

**Published:** 2023-09-19

**Authors:** Lin Zhou, Jianing Zhang, Dan Zhang, Ye Yu, Mengqi Jiang, Huiying Liu, Jiatong Li, Minghui Li, Zhuo Zhang, Lianying Guo

**Affiliations:** ^1^ School of Public Health, Shenyang Medical College, Shenyang, China; ^2^ Department of School Health, Shenyang Center for Disease Control and Prevention, Shenyang, China

**Keywords:** vitamins, co-exposure, diabetes, risk, mortality

## Abstract

**Objective:**

Although the effect of vitamins on the risk of mortality in diabetic patients has been reported, most studies focus on individual vitamins. However, humans are often exposed to multiple vitamins simultaneously in daily life. Therefore, it is worth exploring the effects of co-exposure to multiple vitamins on the risk of mortality in diabetic patients.

**Methods:**

This study included diabetic patients aged ≥20WD years who participated in NHANES from 2003 to 2006. An unsupervised K-means clustering method was used to cluster eight vitamins in serum into several patterns of co-exposure to multiple vitamins, and the Cox proportional hazards model was used to evaluate the impact of different patterns of co-exposure to multiple vitamins on the risk of all-cause mortality in diabetic patients.

**Results:**

Three patterns of co-exposure to multiple vitamins were generated based on K-means clustering, namely, low-level, moderate-level, and high-level. Among the 484 diabetic patients, with a median follow-up of 13.7 years, a total of 211 deaths occurred. After adjusting for covariates, the individual vitamins had varying effects on the risk of all-cause mortality in diabetic patients. Compared to the low-level group of co-exposure to multiple vitamins, the high-level group significantly reduced the risk of all-cause mortality in diabetic patients, with a HR of 0.42 (95% CI: 0.20, 0.87). Subgroup analysis demonstrated that high levels of co-exposure to multiple vitamins significantly reduced the risk of all-cause mortality in males, individuals aged ≥ 60 years, and non-Hispanic White people with diabetes compared to the low-level group, with HR of 0.42 (95% CI: 0.18, 0.98), 0.53 (95% CI: 0.26, 0.98), and 0.26 (95% CI: 0.12, 0.58) respectively.

**Conclusion:**

While individual vitamins had different effects on the risk of all-cause mortality in patients with diabetes, high-level co-exposure to multiple vitamins significantly reduced the risk of all-cause mortality in patients with diabetes, with differences observed among genders, ages, and race. This suggests that when developing vitamin intervention strategies for patients with diabetes, consideration should be given not only to the dosage of individual vitamins but also to the variations between different population groups.

## 1 Introduction

Diabetes is a metabolic disorder that is characterized by hyperglycemia as a result of insufficient insulin secretion or inadequate response to insulin in the body. It affects various systems of the body, such as the circulatory, urinary, endocrine, nervous systems, etc. (1) According to a recent report, in 2021, there were an estimated 529 million people with diabetes worldwide (95% UI 500-564 million), with an age-standardized prevalence of 6.1% (95% UI 5.8-6.5%), of which 96.0% were type 2 diabetes. It was estimated that there were 485 million cases aged 20-79 years with diabetes globally and the age-standardized prevalence of diabetes exceeded 10% in 43 countries/regions worldwide (2). According to the International Diabetes Federation Diabetes Atlas, an estimated 6.7 million deaths in adults aged 20-79 years were attributed to diabetes or its complications in 2021, accounting for 12.2% of all deaths in that age group (3). It is evident that diabetes has become a major health problem worldwide and more measures are needed to prevent or reduce the occurrence of diabetes and its complications.

Vitamins, as coenzymes or antioxidants involved in the metabolism of substances in the body, can have an impact on the occurrence and development of diabetes when there is a deficiency or imbalance. There have been numerous reports on the effects of individual vitamins on diabetes and its complications. For example, a review by Iqbal et al. (4) suggested that vitamin A supplementation delayed the onset of type 2 diabetes in populations with vitamin A deficiency, and a meta-analysis by Li et al. (5) showed that vitamin D supplementation raised the level of 25-hydroxyvitamin D3 in serum and reduced insulin resistance in populations with vitamin D deficiency at baseline. Mascolo et al. (6) pointed out that vitamin B6 deficiency was both a consequence and a cause of diabetes. In addition, Mason et al. (7) found that vitamin C acted as a potent antioxidant *in vivo* and that vitamin C supplementation may contribute to glycemic control and reduce the risk of cardiovascular disease in individuals with type 2 diabetes. However, a prospective study by Eshake et al. (8) found that after a 5-year follow-up, dietary intake of vitamins K and E was associated with a reduced risk of developing diabetes, while no association was observed with the intake of vitamins A or D. Pittas et al. (9) conducted a study that showed daily supplementation of 4000 IU of vitamin D3 did not significantly lower the risk of diabetes compared to a placebo in a high-risk population for type 2 diabetes who were not selected based on vitamin D deficiency.

Recent reports have also discussed the impact of individual vitamins on the risk of mortality in patients with diabetes. Wan et al. (10) investigated the association between serum 25-hydroxyvitamin D levels and all-cause mortality and cause-specific mortality in patients with diabetes and the results revealed that higher serum 25-hydroxyvitamin D levels were significantly associated with lower rates of all-cause mortality and cardiovascular disease-related mortality. Liu et al. (11) reported that serum folate and vitamin B12 levels were associated with cardiovascular disease mortality in patients with type 2 diabetes and the findings suggested that maintaining adequate serum folate and vitamin B12 levels may reduce the risk of cardiovascular disease mortality in patients with type 2 diabetes. Qiu et al. (12) examined the association between serum carotenoid levels and the risk of cardiovascular disease mortality in patients with type 2 diabetes and found that higher serum β-carotene concentrations were significantly correlated with increased risk of cardiovascular mortality in diabetic patients. However, Peng et al. (13) investigated the relationship between serum nutritional biomarkers and the risk of all-cause mortality and cause-specific mortality in individuals with metabolic syndrome and the results indicated that α-carotene may be the primary protective biomarker against mortality risk in individuals with metabolic syndrome, while folate and vitamin B12 were not associated with mortality risk in metabolic syndrome population.

In summary, although the effects of vitamins on diabetes have been reported, most studies focused on individual vitamins as factors, and the results are not entirely consistent. However, in daily life, humans are often exposed to multiple vitamins at the same time and there may be interactions between them (14, 15). It remains unknown whether co-exposure to multiple vitamins plays a greater role in reducing the risk of mortality in patients with diabetes. In this study, large-scale survey data from the National Health and Nutrition Examination Survey (NHANES) in the United States was employed and participants aged≥20 years with diabetes who participated in the NHANES from 2003 to 2006 were finally included as the investigated subjects to explore the association between co-exposure to multiple vitamins and the risk of mortality in patients with diabetes, filling the gap and providing scientific and effective guidance for the reduction of diabetes mortality.

## 2 Methods

### 2.1 Study design and participants

This was a prospective study and consisted of diabetes aged ≥20 who participated in the 2003-2004 and 2005-2006 NHANES. NHANES is a periodic large-scale cross-sectional survey conducted by the National Center for Health Statistics (NCHS) in the United States, which collects data on residents’ health and nutritional status, including adults and children, every two years. A complex, multi-stage, and probability sampling design was used to collect representative samples of residents by NHANES and participants provided information on demographic and socio-economic characteristics, health-related behaviors, and health status using standardized questionnaires. The questionnaires were collected by trained interviewers during recruitment for the study and physical measurements and laboratory tests were also performed by trained medical professionals at mobile examination centers. The follow-up time was calculated based on the date of physical examination and the date of death due to diabetes or the end date of the follow-up period. Mortality status in NHANES was ascertained by linking to the National Death Index as of December 31, 2019.

**Figure 1:**
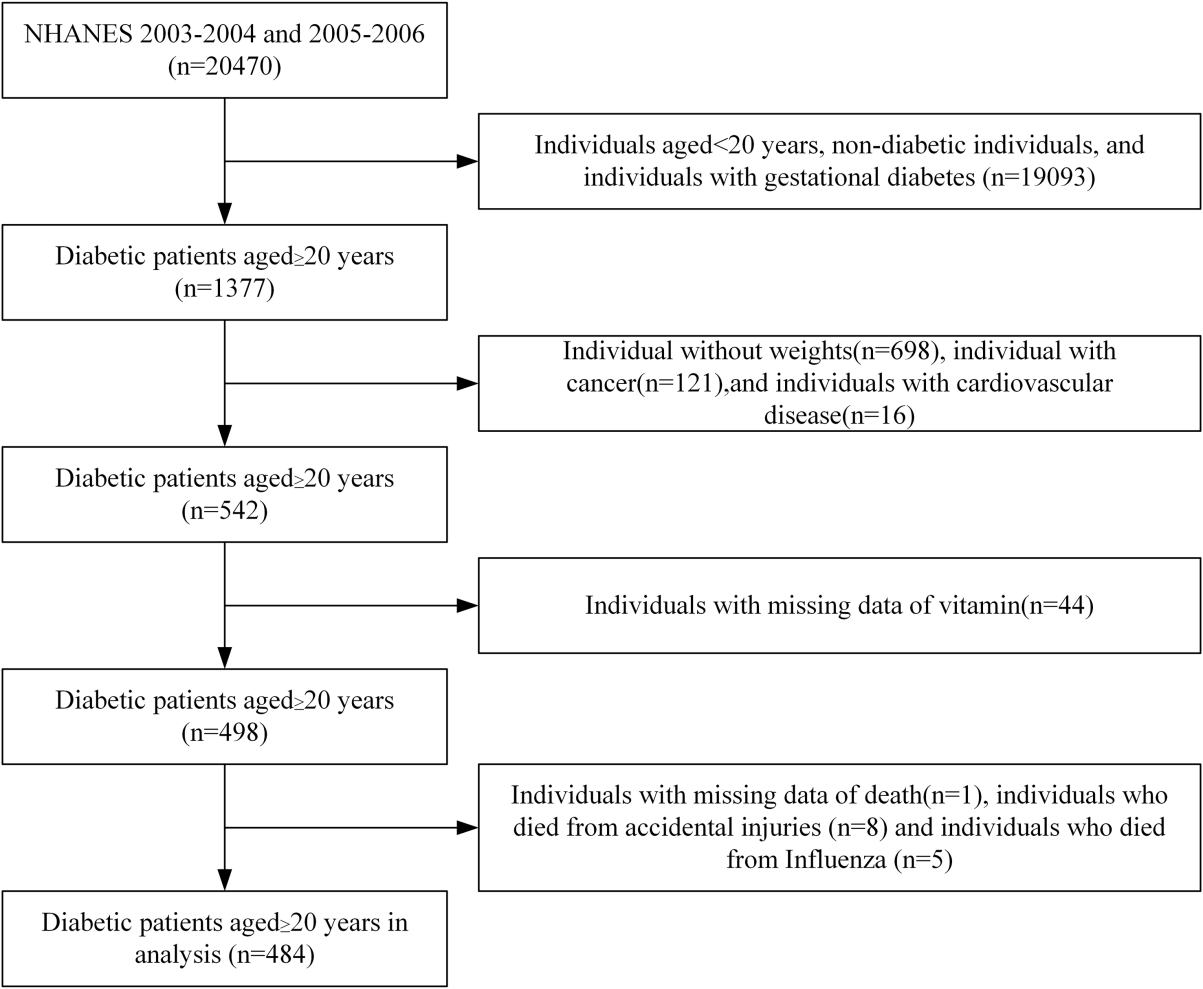
Flow chart for selection of participants.

Inclusion criteria: Diabetic patients aged 20 years and above who participated in the NHANES surveys conducted in 2003-2004 and 2005-2006 in the United States. Exclusion criteria: individuals with gestational diabetes; individuals with missing data on weight, serum vitamin levels, and mortality; individuals diagnosed with cancer at the time of investigation; individuals with cardiovascular diseases before developing diabetes; individuals who died from accidental injuries and influenza. In the end, a total of 484 participants were included in the study (**Figure 1**).

The NHANES program received approval from the Research Ethics Review Committee of the US National Center for Health Statistics, and all participants were informed and gave their consent. More details about the NHANES survey design, methods, and data collection can be obtained from its official website (https://wwwn.cdc.gov/nchs/nhanes/Default.aspx). The study was based on open-source data and research involving public data was approved by the Ethics Committee of the Shenyang Center for Disease Control and Prevention (sycdc-2023-001).

### 2.2 Diagnostic criteria for diabetes

The diagnostic criteria for diabetes included meeting one of the following conditions: 1) self-reported diagnosis of diabetes by a doctor; 2) self-reported use of diabetes medication or insulin injections; 3) according to the American Diabetes Association guidelines, a fasting blood glucose level ≥7mmol/L (or 126 mg/dL), or a glycated hemoglobin level ≥6.5%, or a two-hour blood glucose level ≥ 200 mg/dL in an oral glucose tolerance test.

### 2.3 Measurement of serum vitamins

Blood samples were drawn from participants in mobile examination centers and processed, frozen, stored at -20°C, and transported to the National Center for Environmental Health for analysis. Serum vitamin A (retinol), vitamin E (α-tocopherol), and carotene (β-carotene) were measured using high-performance liquid chromatography (HPLC) with multi-wavelength photodiode array absorbance detection. Serum vitamin C and vitamin B6 were measured using HPLC. Both serum folate and vitamin B12 were measured by using the “Quantaphase II Folate/Vitamin B12” radioassay kit (Bio-Rad Laboratories, America). Serum 25-hydroxyvitamin D was measured using the radioimmunoassay kit (DiaSorin, Italy). All laboratory measurement information is publicly available on the NHANES website.

### 2.4 Assessment of covariates

The covariates in this study included age, sex (male or female), race (non-Hispanic White people, non-Hispanic Black people, other race), education level (less than high school, high school, college), marital status (married or other), poverty income ratio (PIR) (≤ 1.0, 1.0-3.0, >3.0), physical activity (low intensity, moderate intensity, high intensity), smoking status (never smoked, formerly smoked; currently smoked), alcohol consumption (no, low to moderate, heavy), body mass index (BMI) (<25, 25.0-29.9, ≥30.0), hypertension (yes or no), high cholesterol (yes or no), liver disease (yes or no), kidney disease (yes or no), and healthy eating index-2015 (HEI-2015). The “other” category in marital status referred to individuals who were unmarried, divorced, widowed, or married but separated. Physical activity was based on self-reported exercise intensity in the past month. Alcohol consumption was defined based on self-reported average daily drinking amount, where low to moderate drinkers were described as consuming less than 2 glasses per day for men and less than 1 glass per day for women, and heavy drinkers were defined as men consuming 2 or more glasses per day for men and 1 or more glasses per day for women. BMI was calculated as weight (in kilograms) divided by height (in meters) squared and hypertension, high cholesterol, liver disease, and kidney disease were self-reported as having been diagnosed by a doctor. HEI-2015 scores were calculated from a one-day, 24-hour retrospective survey interview. More details on these covariates can be obtained from the NHANES website.

### 2.5 Statistical analysis

Referring to the analysis guidelines of the NHANES survey, sampling weights were used in the analysis. Quantitative data were tested for normality and expressed as mean ± standard deviation if normally distributed, and ANOVA was conducted for comparisons between multiple groups. If the data was not normally distributed, it was expressed as median (interquartile range, IQR), and group comparisons were conducted using the Wilcoxon rank-sum test. For qualitative data, the number of cases (percentage) was described and the chi-square test was used for statistical testing. The study involved 8 common vitamins, and K-means clustering was performed on these 8 vitamins. Before the clustering analysis, logarithmic transformation was applied to serum vitamin measurements with severe skewness, followed by normalization to eliminate the influence of various units.

A dose-response analysis of the association between individual vitamin levels and the risk of mortality in patients with diabetes was performed using the restricted cubic spline (RCS). Cox proportional hazards models were used to calculate the hazard ratios (HR) and 95% confidence intervals (CI) for the association between individual serum vitamin levels, as well as the multivitamin co-exposure levels, and the risk of mortality in patients with diabetes. Four models were fitted, with model 1 when unadjusted for covariates, model 2 when adjusted for age, sex, education, and income level, model 3 when further adjusted for physical activity, smoking, alcohol consumption, BMI, and diet, and model 4 when further adjusted for hypertension, high cholesterol, liver disease, and kidney disease. We performed stratified analyses based on age (<60 years or ≥60 years), sex, and race. Potential modifying effects were examined by testing the corresponding multiplicative interaction terms.

Two sensitivity analyses were conducted to test the robustness of the results. In one sensitivity analysis, Cox proportional hazards analysis was performed without weighting the data to assess the effect of weighting on the results. Regarding another sensitivity analysis, the follow-up time was adjusted by linking the mortality data to the national mortality index as of December 31, 2015, to evaluate the influence of follow-up duration on the results.

All analyses were performed using R 4.2.2 software and statistical analyses were conducted as two-sided tests. The significance level was set at a *P* value < 0.05.

## 3 Result

### 3.1 Cluster analysis of serum vitamins in patients with diabetes

**Figure 2:**
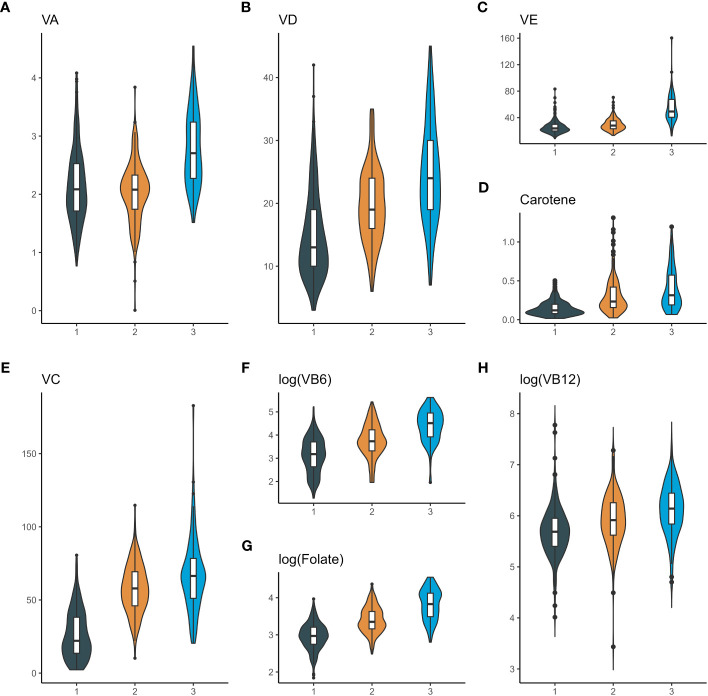
Violin plots of serum vitamin concentrations in different clusters of diabetic patients. **(A)**, violin plots of serum vitamin A; **(B)**, violin plots of serum vitamin D; **(C)**, violin plots of serum vitamin E; **(D)**, violin plots of serum Carotene; **(E)**, violin plots of serum vitamin C; **(F)**, violin plots of serum vitamin B6; **(G)**, violin plots of serum Folate; **(H)**, violin plots of serum vitamin B12. The X-axis represents three clusters: 1. Cluster 1 (low-level group with co-exposure to multiple vitamins); 2. Cluster 2 (moderate-level group with co-exposure to multiple vitamins); 3. Cluster 3 (high-level group with co-exposure to multiple vitamins). The Y-axis represents serum vitamin levels. VA, vitamin A; VD, vitamin D; VE, vitamin E; VC, vitamin C; VB6, vitamin B6; VB12, vitamin B12.

Eight serum vitamins in 484 diabetics were clustered into three clusters using the K-means clustering method (**Supplementary Figure 1**). The levels of these three clusters of vitamins, expressed as medians (interquartile range), were listed in **Table 1** and visualized in **Figure 2**. According to **Table 1** and **Figure 2**, it was observed that the first cluster exhibited the lowest levels of all vitamins in the serum, which was referred to as the low-level group with co-exposure to multiple vitamins. In the second cluster, except for the vitamin A level which was similar to that of the first cluster, the other vitamins had moderate levels and therefore the group was regarded as the moderate-level group with co-exposure to multiple vitamins. The third cluster had the highest levels of each vitamin in the serum and was termed the high-level group with co-exposure to multiple vitamins.

**Table 1:** Serum vitamin levels of three clusters in diabetic patients.

Characteristic	Overall, N = 484^1^	Cluster 1, N = 208^1^	Cluster 2, N = 187^1^	Cluster 3, N = 89^1^
VA (μmol/L)	2.16 (1.81, 2.58)	2.09 (1.71, 2.53)	2.08 (1.74, 2.33)	2.71 (2.27, 3.24)
VD (ng/mL)	27.9 (21.9, 37.5)	23.7 (19.8, 29.8)	27.9 (23.2, 35.1)	49.2 (40.5, 67.5)
VE (μmol/L) ^a^	0.19 (0.10, 0.33)	0.12 (0.08, 0.20)	0.23 (0.16, 0.42)	0.31 (0.19, 0.57)
Carotene (μmol/L) ^b^	18.0 (13.0, 24.0)	13.0 (10.0, 19.0)	19.0 (16.0, 24.0)	24.0 (19.0, 30.0)
VC (μmol/L)	46.0 (26.0, 63.2)	22.1 (13.6, 38.2)	57.9 (46.0, 69.3)	66.4 (51.1, 78.4)
Log (VB6) (nmol/L)	3.65 (3.07, 4.20)	3.18 (2.63, 3.70)	3.73 (3.32, 4.23)	4.51 (3.92, 4.95)
Log (Folate) (nmol/L)	3.25 (2.97, 3.60)	2.97 (2.75, 3.21)	3.35 (3.16, 3.63)	3.83 (3.49, 4.12)
Log (VB12) (pmol/L)	5.84 (5.53, 6.17)	5.69 (5.40, 5.95)	5.91 (5.62, 6.26)	6.14 (5.84, 6.44)

^1^Median (IQR).

a serum vitamin E levels are expressed in terms of alpha-tocopherol levels.

b serum carotene levels are expressed in terms of beta-carotene levels.

Cluster 1, low-level group with co-exposure to multiple vitamins; Cluster 2, moderate-level group with co-exposure to multiple vitamins; Cluster 3, high-level group with co-exposure to multiple vitamins. VA, Vitamin A; VD, Vitamin D; VE, Vitamin E; VC, Vitamin C; VB6, Vitamin B6; VB12, Vitamin B12.

### 3.2 Baseline characteristics of participants

**Table 2** presented the baseline characteristics of 484 participants in this study, with a median age of 57.0 years, under three different patterns of co-exposure to multiple vitamins. The proportion of males slightly exceeded that of females, accounting for 52% and 48% respectively. Non-Hispanic White people had the highest percentage among races (38%), followed by other races (34%), while non-Hispanic Black people had the lowest percentage (28%). The covariates of age, race, marital status, educational level, BMI, smoking, insulin, albumin, aspartate aminotransferase, and blood urea nitrogen were unevenly distributed among the three groups.

**Table 2:** Baseline characteristics of participants in this study.

Characteristic	Overall, N = 484 (100%)^1^	Cluster 1, N = 208 (43%)^1^	Cluster 2, N = 187 (39%)^1^	Cluster 3, N = 89 (18%)^1^	*P* Value^2^
Age (years)	57.0 (46.0, 67.0)	52.0 (43.8, 63.4)	57.0 (46.2, 70.0)	65.4 (56.0, 71.0)	<0.001
Sex					0.120
Male	254 (52%)	112 (54%)	104 (56%)	38 (43%)	
Female	230 (48%)	96 (46%)	83 (44%)	51 (57%)	
Race					<0.001
Non-Hispanic White people	184 (38%)	71 (34%)	65 (35%)	48 (54%)	
Non-Hispanic Black people	135 (28%)	79 (38%)	41 (22%)	15 (17%)	
Other	165 (34%)	58 (28%)	81 (43%)	26 (29%)	
Marital status					0.018
Married	291 (60%)	111 (53%)	126 (67%)	54 (61%)	
Other	193 (40%)	97 (47%)	61 (33%)	35 (39%)	
Educational level					0.031
Below high school	110 (23%)	41 (20%)	54 (29%)	15 (16%)	
High School	211 (44%)	103 (49%)	71 (38%)	37 (42%)	
College or above	163 (33%)	64 (31%)	62 (33%)	37 (42%)	
PIR					0.120
≤1.0	92 (19%)	48 (23%)	35 (19%)	9 (10%)	
1.0-3.0	271 (56%)	(54%)	104 (56%)	56 (63%)	
>3.0	92 (19%)	32 (15%)	38 (20%)	22 (25%)	
Miss	29 (6%)	17 (8%)	10 (5%)	2 (2%)	
Active					0.120
Low	264 (55%)	131 (63%)	85 (46%)	48 (54%)	
Moderate	158 (33%)	62 (30%)	70 (37%)	26 (29%)	
High	62 (12%)	15 (7%)	32 (17%)	15 (17%)	
BMI (kg/m^2^)					<0.001
< 25	65 (13%)	13 (6%)	34 (18%)	18 (20%)	
25.0-29.9	131 (27%)	50 (24%)	46 (25%)	35 (39%)	
≥30.0	275 (57%)	137 (66%)	104 (55%)	34 (38%)	
Miss	13 (3%)	8 (4%)	3 (2%)	2 (3%)	
Alcohol					0.500
None	173 (36%)	77 (37%)	59 (32%)	37 (42%)	
Low-to-Moderate	192 (40%)	75 (36%)	82 (44%)	35 (39%)	
Heavy	88 (18%)	43 (21%)	34 (18%)	11 (12%)	
Miss	31 (6%)	13 (6%)	12 (6%)	6 (7%)	
Smoking					<0.001
Never smoke	226 (47%)	83 (40%)	98 (53%)	45 (51%)	
Former smoker	163 (33%)	61 (29%)	66 (35%)	36 (40%)	
Current smoker	95 (20%)	64 (31%)	23 (12%)	8 (9%)	
Hypertension					0.200
Yes	312 (64%)	140 (67%)	112 (60%)	60 (67%)	
No	172 (36%)	68 (33%)	75 (40%)	29 (33%)	
High Cholesterol					0.056
Yes	242 (50%)	94 (45%)	90 (48%)	58 (65%)	
No	163 (34%)	73 (35%)	66 (35%)	24 (27%)	
Miss	79 (16%)	41 (20%)	31 (17%)	7 (8%)	
Liver disease					0.200
Yes	12 (2%)	6 (3%)	5 (3%)	1 (1%)	
No	9 (2%)	2 (1%)	3 (2%)	4 (5%)	
Miss	463 (96%)	200 (96%)	179 (95%)	84 (94%)	
Kidney disease					0.600
Yes	42 (9%)	18 (9%)	14 (7%)	10 (11%)	
No	442 (91%)	190 (91%)	173 (93%)	79 (89%)	
HEI-2015	52.0 (41.4, 62.5)	47.0 (37.5, 57.0)	56.0 (45.0, 67.5)	54.0 (44.0, 65.1)	0.13
Glucose (mg/dL)	135.0(119.2, 168.0)	134.0(114.9, 179.6)	135.9(123.4, 162.0)	134.5(116.5, 161.1)	0.5
Insulin (uU/mL)	12.3 (6.9, 21.6)	14.4 (10.0, 24.1)	11.8 (6.2, 21.6)	9.0 (5.7, 13.5)	<0.001
Glycohemoglobin (%)	6.5 (6.0, 7.6)	6.6 (5.9, 7.9)	6.5 (6.0, 7.5)	6.5 (5.9, 7.4)	0.5
Albumin (g/dL)	4.1 (3.9, 4.3)	4.0 (3.8, 4.2)	4.1 (3.9, 4.3)	4.2 (4.0, 4.4)	<0.001
Total protein (g/dL)	7.1 (6.8, 7.4)	7.0 (6.8, 7.3)	7.2 (6.9, 7.5)	7.1 (6.8, 7.3)	0.2
GGT (U/L)	25.0 (18.0, 38.0)	26.0 (20.0, 40.8)	26.0 (17.0, 37.2)	21.4 (15.4, 34.3)	0.3
ALT (U/L)	23.0 (18.0, 31.0)	23.0 (18.0, 30.0)	24.0 (17.0, 34.0)	22.0 (19.0, 26.5)	0.2
AST (U/L)	23.0 (19.0, 28.0)	22.0 (18.0, 26.0)	24.0 (20.0, 31.0)	23.4 (20.0, 28.0)	<0.001
Blood urea nitrogen (mg/dL)	13.0 (10.0, 17.0)	12.0 (10.0, 16.0)	13.0 (11.0, 17.0)	15.5 (12.0, 20.0)	0.011
Creatinine (μmol/L)	79.6 (61.9, 88.4)	79.6 (70.7, 97.2)	70.7 (61.9, 88.4)	70.7 (61.9, 97.2)	0.14

^1^median (IQR) for continuous; n (%) for categorical.

^2^Wilcoxon rank-sum test for complex survey samples; chi-squared test with Rao & Scott’s second-order correction.

PIR, poverty income ratio; BMI, body mass index; HEI-2015, Healthy Eating Index-2015; GGT, gamma-glutamyl transferase; ALT, alanine aminotransferase; AST, aspartate aminotransferase. Cluster 1, low-level group with co-exposure to multiple vitamins; Cluster 2, moderate-level group with co-exposure to multiple vitamins; Cluster 3, high-level group with co-exposure to multiple vitamins.

### 3.3 Cox proportional hazards analysis of individual vitamins and all-cause mortality in diabetic patients

**Figure 3:**
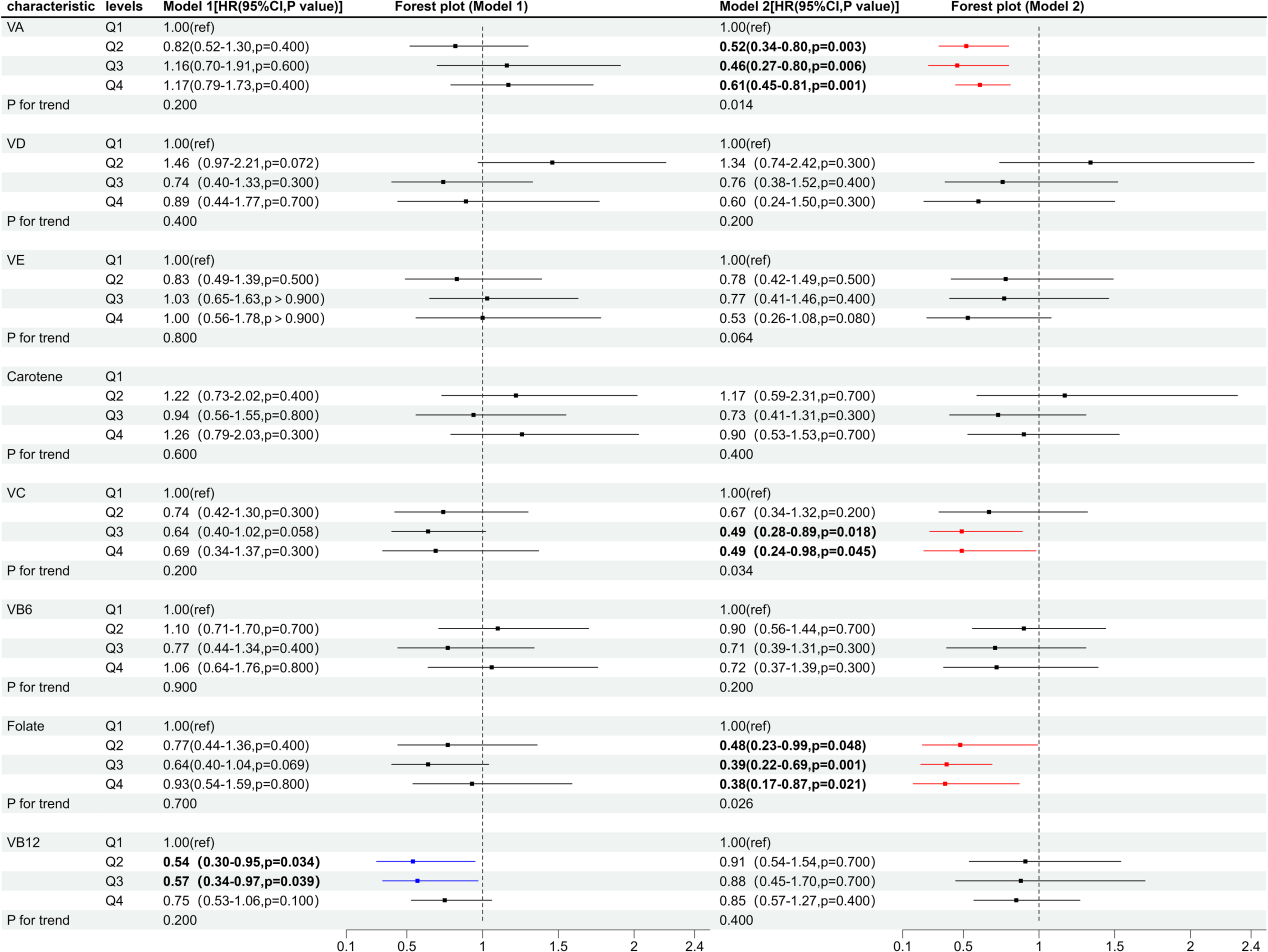
Forest plot of individual vitamins and the risk of all-cause mortality in diabetic patients. Q1, first quartile; Q2, second quartile; Q3, third quartile; Q4, fourth quartile. Model 1, unadjusted for covariates; Model 2, adjusted for age, gender, marital status, education level, PIR, smoking, alcohol consumption, physical activity, BMI, HEI-2015, hypertension, high cholesterol, liver disease, and kidney disease. VA, Vitamin A; VD, Vitamin D; VE, Vitamin E; VC, Vitamin C; VB6, Vitamin B6; VB12, Vitamin B12.

The median follow-up of the 484 diabetic patients in this investigation was 13.7 years, during which a total of 211 deaths were reported, including 75 from cardiac diseases, 36 from malignant tumors, 6 from chronic lower respiratory diseases, 11 from cerebrovascular diseases, 9 from Alzheimer’s disease, 26 from diabetes mellitus, 7 from Nephritis, nephrotic syndrome and kidney disease and 41 from all other causes. Given that the contribution of individual vitamins to the risk of mortality in diabetic patients may vary, the relationship between individual vitamins and the risk of all-cause mortality in diabetic patients was analyzed. The individual vitamins were divided into quartiles: the first quartile (Q1) group, the second quartile (Q2) group, the third quartile (Q3) group, and the fourth quartile (Q4) group. As shown in **Figure 3**, after adjusting for covariates, vitamin A and folate significantly reduced the risk of all-cause mortality in patients with diabetes at Q2, Q3, and Q4 levels compared to the Q1 level. The HR of Q2, Q3, and Q4 levels were 0.52 (95%CI: 0.34, 0.80), 0.46 (95%CI: 0.27, 0.80), and 0.61 (95%CI: 0.45, 0.81) for vitamin A, and 0.48 (95%CI: 0.23, 0.99), 0.39 (95%CI: 0.22, 0.69), and 0.38 (95%CI: 0.17, 0.87) for folate. Vitamin C at the Q3 and Q4 levels also significantly decreased the risk of all-cause mortality in diabetic patients, with HRs of 0.49 (95% CI: 0.28, 0.89), and 0.49 (95% CI: 0.24, 0.98), respectively. However, no significant associations were observed between other vitamins and the risk of all-cause mortality in diabetic patients.

### 3.4 Dose-response of individual vitamins on the risk of all-cause mortality in diabetic patients

**Figure 4:**
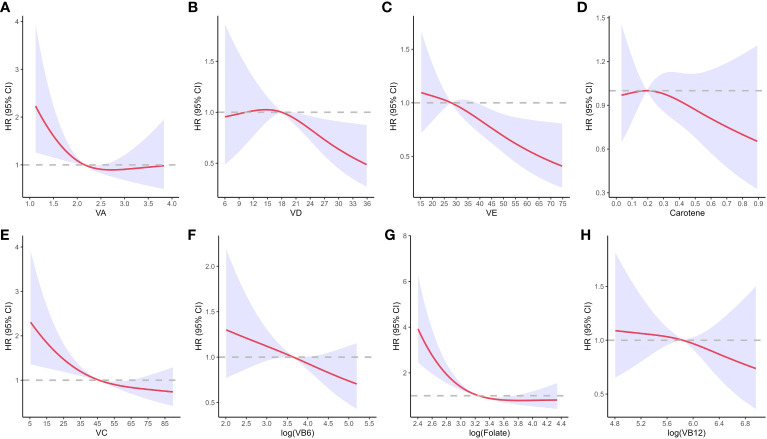
Dose-response of individual vitamins on the risk of all-cause mortality in diabetic patients. **(A)**, dose-response of vitamin A; **(B)**, dose-response of vitamin D; **(C)**, dose-response of vitamin E; **(D)**, dose-response of carotene; **(E)**, dose-response of vitamin C; **(F)**, dose-response of vitamin B6; **(G)**, dose-response of Folate; **(H)**, dose-response of vitamin B12. The X-axis represents the serum vitamin levels, and the y-axis represents the adjusted hazard ratio (HR, 95% confidence interval) for all-cause mortality in diabetic patients after adjusting for age, gender, marital status, education level, PIR, smoking, alcohol consumption, physical activity, BMI, HEI-2015, hypertension, high cholesterol, liver disease, and kidney disease. VA, Vitamin A; VD, Vitamin D; VE, Vitamin E; VC, Vitamin C; VB6, Vitamin B6; VB12, Vitamin B12.

The RCS analysis results (**Figure 4**) suggested that low levels of vitamin A, vitamin C, and folate in the serum were risk factors for all-cause mortality in diabetic patients and the risk of mortality decreased with increasing serum levels of each vitamin. Serum levels of vitamin A at 2.17-2.50 μmol/L, vitamin C at 46.4-63.8 μmol/L and log levels of folate at 3.26-3.74 nmol/L significantly reduced the risk of all-cause mortality in diabetic patients. However, as the levels further increased, they failed to lower the risk of all-cause mortality in diabetic patients and on the contrary, higher levels of vitamin A showed a trend of increasing the risk of all-cause mortality in diabetic patients. Serum vitamin D and vitamin E were not associated with the risk of all-cause mortality in diabetic patients at low levels but significantly reduced the risk of all-cause mortality in diabetic patients when vitamin D levels were ≥20.8 ng/mL, vitamin E levels were ≥38.9 μmol/L. In addition, the levels of carotene, vitamin B6, and vitamin B12 in the serum were not associated with the risk of all-cause mortality in diabetic patients.

### 3.5 Association between co-exposure of multiple vitamins and the risk of all-cause mortality in diabetic patients

**Figure 5:**
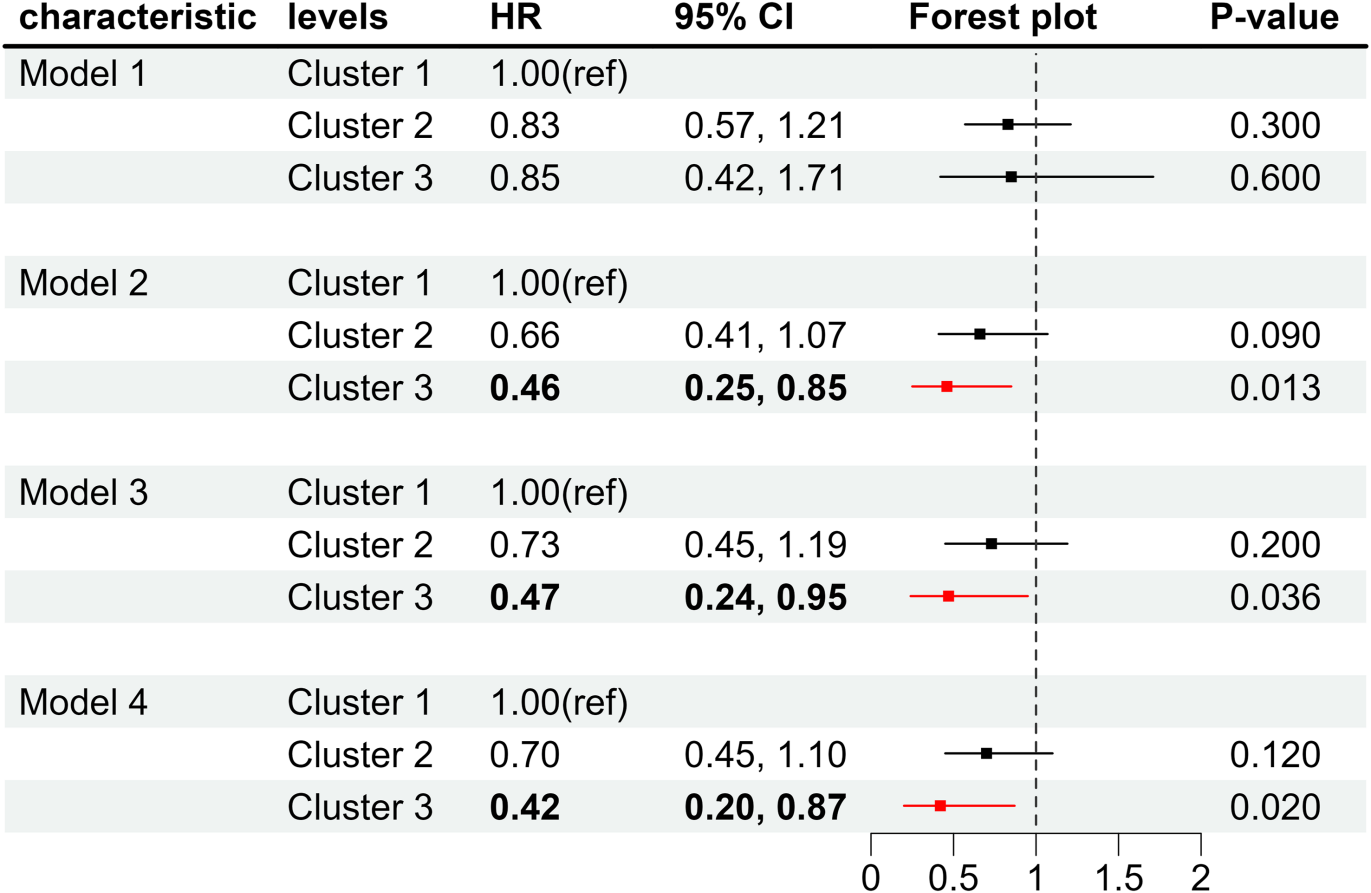
Forest plot of the co-exposure of multiple vitamins and the risk of all-cause mortality in diabetic patients. Cluster 1, low-level group with co-exposure to multiple vitamins; Cluster 2, moderate-level group with co-exposure to multiple vitamins; Cluster 3, high-level group with co-exposure to multiple vitamins. Model 1, unadjusted for covariates; Model 2, adjusted for age, gender, marital status, education level, and PIR; Model3, further adjusted for physical activity, smoking, alcohol consumption, BMI, and HEI-2015; Model 4, further adjusted for hypertension, high cholesterol, liver disease, and kidney disease.

As shown in **Figure 5**, compared with the low-level group of co-exposure to multiple vitamins, the HR of all-cause mortality in the high-level group of diabetes was 0.46 (95%CI: 0.25, 0.85) after adjusting for age, sex, marital status, education level and income. When further adjusting for physical activity, smoking status, alcohol consumption, BMI, and HEI-2015, the HR of all-cause mortality in the high-level group was 0.47 (95% CI: 0.24, 0.95). Moreover, the HR of all-cause mortality in the high-level group was 0.42 (95%CI: 0.20, 0.87) after further adjusting for hypertension, high cholesterol, liver disease and kidney disease. These results indicated that regardless of the adjustment for covariates, the high-level group could significantly reduce the risk of all-cause mortality in diabetes compared with the low-level group.

### 3.6 Subgroup analysis

**Figure 6:**
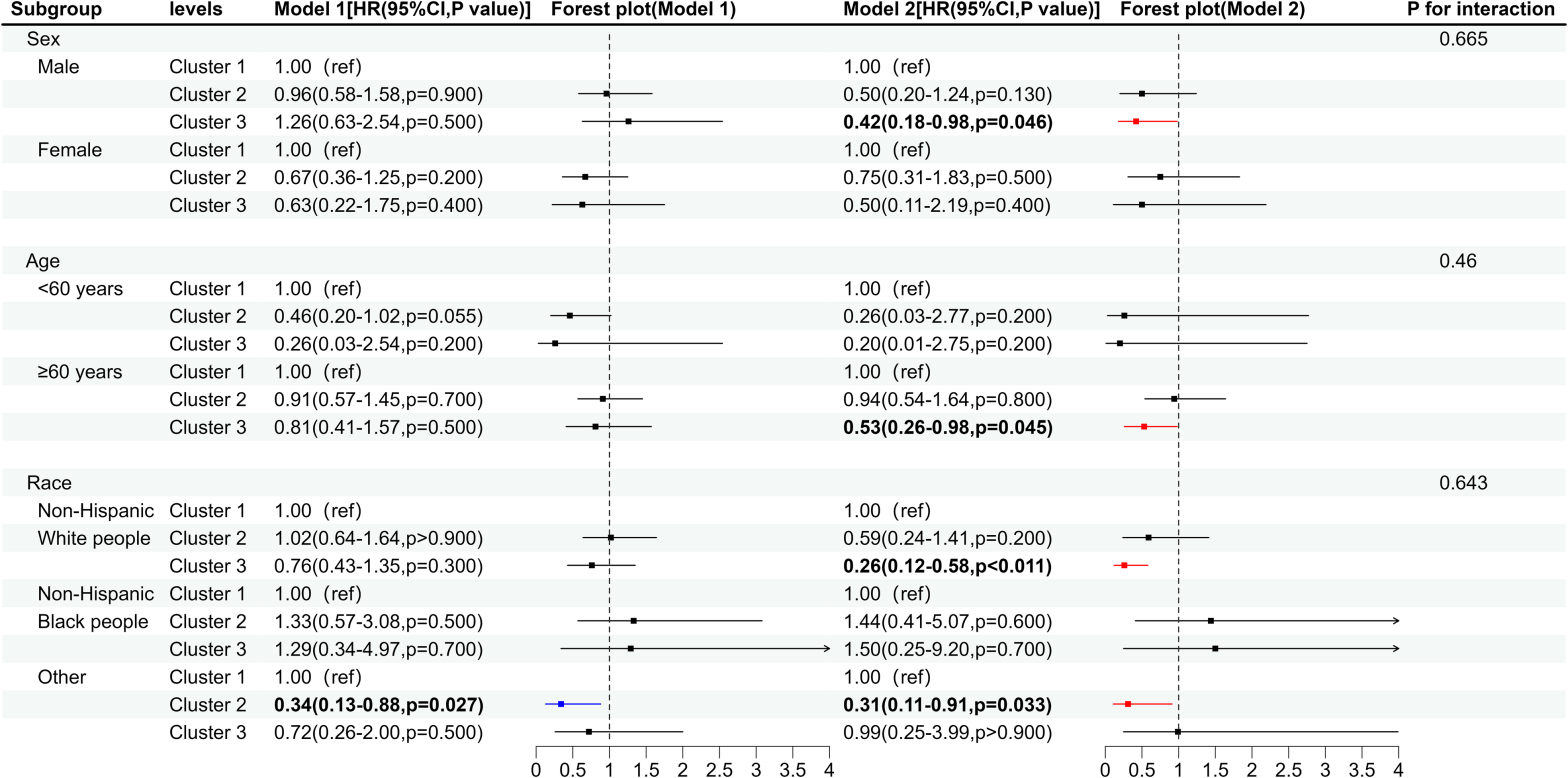
Forest plot of the co-exposure of multiple vitamins and the risk of all-cause mortality in subgroups of diabetic patients. Cluster 1, low-level group with co-exposure to multiple vitamins; Cluster 2, moderate-level group with co-exposure to multiple vitamins; Cluster 3, high-level group with co-exposure to multiple vitamins. Model 1, unadjusted for covariates; Model 2, adjusted for age, gender, marital status, education level, PIR, smoking, alcohol consumption, physical activity, BMI, HEI-2015, hypertension, high cholesterol, liver disease, and kidney disease.

As can be seen from **Figure 6**, after adjusting for covariates, compared to the low-level group with co-exposure to multiple vitamins, the high-level groups showed a significant reduction in the risk of all-cause mortality among male diabetes patients, with HR of 0.42 (95% CI: 0.18, 0.98), whereas no such effect was observed in females. Among individuals aged ≥ 60 years, the high-level group had a significantly lower risk of all-cause mortality among diabetes patients, with a HR of 0.53 (95% CI: 0.26, 0.98). In terms of race, the co-exposure to high-level multiple vitamins reduced the risk of all-cause mortality among non-Hispanic White people with diabetes, with a HR of 0.26 (95% CI: 0.12, 0.58), while co-exposure to moderate-level multiple vitamins lowered the risk of all-cause mortality among diabetic individuals of other race, with a HR of 0.31 (98% CI: 0.11, 0.91).

### 3.7 Sensitivity analysis

Cox proportional hazards analysis was performed without weighting the data, and no significant changes were observed in the results (**Supplementary Table 1**). Adjusting the mortality data until December 31, 2015, with a total of 489 diabetic patients, a median follow-up of 10.2 years, and 152 deaths. The results of the Cox proportional hazards analysis showed no significant change (**Supplementary Table 2**), indicating the robustness of the findings.

## 4 Discussion

In 2021, approximately 6.7 million adults aged 20-79 died from diabetes or its complications, accounting for 12.2% of overall mortality in this age group (3). With such a high disease burden of diabetes, exploring factors that can reduce the risk of diabetes-related mortality becomes increasingly important. In this study, an unsupervised K-means clustering method was used to classify the eight vitamins into three groups of vitamin patterns with different concentrations among representative US adults to explore the effect of co-exposure to multiple vitamins on the risk of mortality in diabetic patients. After adjusting for various covariates, we found that the high-level group with co-exposure to multiple vitamins was associated with a reduced risk of diabetes-related mortality compared to the low-level group, with a HR of 0.42 (95% CI: 0.20, 0.87, *P*=0.02). In addition, since individual vitamins have varying effects on reducing the risk of mortality in diabetic patients, we further analyzed the impact of each vitamin on the risk of diabetes-related mortality.

### 4.1 Individual vitamin and the risk of all-cause mortality in diabetic patients

According to the comprehensive analysis of Cox proportional hazards and dose response, vitamin A, vitamin C, and folate were found to be risk factors for all-cause mortality in diabetic patients when their serum levels were low, which was consistent with the previous studies’ results. Krempf et al. (16) demonstrated that the concentration of vitamin A in the blood of diabetic patients was significantly lower than that of the control group. Sun et al. (17) found that dietary intake of vitamin C below the estimated average requirement (EAR) was associated with a notably increased risk of developing diabetes in adults, and diabetic patients with lower serum vitamin C levels had shorter survival time compared to those with normal serum vitamin C levels. Liu et al. (11) revealed a significant correlation between low levels of serum folate and the risk of cardiovascular disease and all-cause mortality in diabetes patients. However, high levels of serum vitamin A, vitamin C, and folate did not significantly lower the risk of all-cause mortality in diabetic patients. Specifically, when serum vitamin A levels > 2.50 μmol/L, the risk of all-cause mortality in diabetes patients exhibited an upward trend as the serum levels of vitamin A increased. This may be attributed to higher serum levels of these vitamins leading to increased production of oxidative metabolites, which can act as pro-oxidants and cause DNA damage (18). To sum up, the risk of all-cause mortality in diabetic patients was significantly reduced only when vitamin A, vitamin C, and folate levels were in the appropriate range.

In this study, dose-response analysis revealed that high levels of serum vitamin D and vitamin E were significantly associated with a decreased risk of all-cause mortality in diabetes patients. which was inconsistent with some previous findings (19–21). However, Cox proportional hazards analysis did not show that vitamin D and vitamin E significantly reduced the risk of all-cause mortality risk among diabetes patients for vitamin D and vitamin E in comparison to the Q1 group. A previous study suggested that low levels of vitamin D may play an important role in the pathogenesis of type 2 diabetes (22), and this trend was also observed in the dose-response analysis conducted in this study. Moreover, research confirmed that vitamin E could improve blood glucose levels primarily through its antioxidant properties, and low levels of vitamin E were not a risk factor for diabetes (23). This may also be the reason why the levels of vitamin D and vitamin E in the other quartiles, were not associated with the all-cause mortality risk in diabetes patients as compared to Q1.

In addition, no association was observed between carotenoids, vitamin B6, and vitamin B12 and all-cause mortality in diabetes patients. However, it was found that low levels of vitamin B6 were associated with the development of diabetes (24, 25). The dose-response analysis in this study also showed a trend of this association. Therefore, further validation is required to establish the relationship between vitamin B6 and all-cause mortality in diabetes patients. Recently, Qiu et al. (12) and Liu et al. (11) reported an association between serum carotene and vitamin B12 and the risk of cardiovascular disease-related mortality in patients with diabetes, respectively. However, due to the limitations of the data in this study, further investigation into the specific causes of death related to vitamins and diabetes was not conducted. Therefore, more research is needed to clarify the relationship between the risk of death in diabetic patients and serum carotene and vitamin B12.

It is important to note that although fat-soluble vitamins, especially vitamin D and vitamin E, are critical in reducing the risk of all-cause mortality in diabetic patients, and their effects increase with dose, excessive intake of fat-soluble vitamins can be detrimental. Long-term intake of vitamin A above the recommended amount may damage the liver (26) and bones (27). Excessive intake of vitamin D can disrupt calcium metabolism, leading to high blood calcium levels, which causes kidney stones, kidney damage, and adverse effects on the cardiovascular system (28). Excess intake of vitamin E interferes with vitamin K metabolism, affecting coagulation and leading to bleeding tendencies (29), and excessive intake of carotenoids can cause pigment deposition and increase the risk of hypertension (30). Therefore, it is essential to adhere to the recommended intake and avoid long-term high-dose consumption.

### 4.2 Co-exposure to multiple vitamins and risk of all-cause mortality in diabetic patients

In an exploration of the impact of co-exposure to multiple vitamins on the risk of all-cause mortality in diabetic patients, various covariates were adjusted and different models were fitted. All models consistently yielded the same result compared to low-level co-exposure to multiple vitamins, high-level co-exposure to multiple vitamins significantly reduces the risk of all-cause mortality in diabetic patients. The significantly lower risk of all-cause mortality in diabetic patients with high-level co-exposure to multiple vitamins may be attributed to the following mechanisms. First, this may be due to the multi-pathway effect. Studies have shown that different individual vitamins have varying mechanisms in regulating blood glucose or improving diabetic complications. Vitamin A can upregulate the function of antioxidant enzymes in the body, reducing oxidative stress levels in diabetic patients (31), and also promote β-cell regeneration (4). Vitamin D can act directly on pancreatic β-cells, affecting insulin secretion (32, 33), improve insulin sensitivity through vitamin D receptors in insulin-sensitive organs (34), and reduce oxidative stress in patients with diabetes (22). Vitamin E and vitamin C may regulate blood glucose levels through its antioxidant effects (21, 35, 36). Vitamin B6 can attenuate insulin resistance by regulating the gene expression involved in fat synthesis (37), and also decrease the formation of advanced glycation end products (AGEs) (38). Folate and vitamin B12 can ameliorate diabetic complications by reducing elevated homocysteine levels and oxidative stress (39, 40). Therefore, when multiple vitamins are co-exposed, they can simultaneously exert their effects through multiple pathways, resulting in better efficacy. Moreover, this is probably attributed to a synergistic effect. The study by Niki et al. (15) demonstrated a synergistic effect between vitamin C and vitamin E, as well as a potential synergistic effect between vitamin E and β-carotene. Gargarl et al. (41) showed that supplementation of folic acid in type 2 diabetes patients treated with high-dose metformin can improve serum vitamin B12 levels. Additionally, Polizzl et al. (42) revealed that the combination of vitamin B6 with B2 decreased DNA glycosylation in leukocytes of diabetes patients, whereas individual use had no such effect. Therefore, when multiple vitamins are ingested simultaneously, there may be a synergistic effect that potentially promotes each other’s actions. However, no significant effect was found in the group of moderate-level co-exposure to multiple vitamins in our study. Thus, further evidence is needed to demonstrate the potential synergistic effect and the strength of the co-exposure to multiple vitamins.

In this study, co-exposure to multiple vitamins in the moderate-level group had no significant effect on all-cause mortality in patients with diabetes. By examining the data, we discovered that the median serum levels of individual vitamins in the high-level group reached or exceeded the optimal point or range, while in the moderate-level group, the median serum levels of individual vitamins approached or fell below the optimal point or range, which may suggest the importance of serum levels of individual vitamins. In addition, the dose-response analysis indicated that individual vitamin levels in serum had an optimal point or range of impact on all-cause mortality in patients with diabetes.

Subgroup analyses revealed that the impact of co-exposure to multiple vitamins on all-cause mortality in diabetes patients varied by sex, age, and race. The effect was more pronounced for individuals aged ≥60 years, males, and populations of races other than non-Hispanic Black people. It was found that the age and racial composition of participants varied significantly in different clusters. The median age of patients with diabetes in the high-level group with co-exposure to multiple vitamins was 65.4 years, while it was less than 60 years in the other groups. Similarly, non-Hispanic White people had the highest proportion in the group of high-level co-exposure to multiple vitamins, while in the group of low-level co-exposure to multiple vitamins, non-Hispanic Black people had the highest proportion. Therefore, we analyzed the serum vitamin levels in different subgroups of diabetes patients and found that individuals aged ≥60 years generally had higher serum vitamin levels compared to those aged <60 years, with significant increases in vitamin A, vitamin E, carotenoids, vitamin C, vitamin B6, and folate. Serum vitamin levels were higher in non-Hispanic White people with diabetes compared to non-Hispanic Black people with diabetes, with significantly greater levels of vitamin D, vitamin E, vitamin C, and folate, whereas serum vitamin levels in other race groups were higher in the second cluster. It can be observed that varying exposure levels of individual vitamins among different age groups and racial populations may contribute to the differences in outcomes among subgroups. Subgroup results further indicated that individual dosages of vitamins were crucial for achieving better outcomes from co-exposure to multiple vitamins. The higher serum vitamin levels in individuals aged ≥ 60 years with diabetes may be due to their generally stronger health awareness and tendency to consume a diet rich in multiple vitamins and vitamin supplements (43) and the higher serum vitamin levels in non-Hispanic White people could be related to their higher cultural level, health consciousness, and more rational dietary habits (44). Additionally, it has also been reported that non-Hispanic Black people had a 15-20 times higher prevalence of vitamin D deficiency than non-Hispanic White people because the melanin in their skin blocks the ultraviolet radiation required for vitamin D synthesis (45). Additionally, non-Hispanic Black people had a higher likelihood of smoking compared to other racial groups (44). Research suggested that the chemicals in tobacco products may interfere with the absorption and utilization of vitamins, making smokers more prone to vitamin deficiencies (46, 47). In diabetic patients of other races, high levels of co-exposure to multiple vitamins did not significantly reduce the risk of all-cause mortality. Further investigation is needed to ascertain the reasons behind this finding.

Contrary to age and race, the serum individual vitamin levels in male diabetes patients were similar to those in female diabetes patients, but the results were still different, indicating that the effect of co-exposure to multiple vitamins on the risk of all-cause mortality in diabetes patients may be influenced not only by the exposure levels but also by the body’s metabolism. Multiple studies have shown significant differences between males and females in terms of both the incidence of diabetes and its complications, which may be attributed to differences in sex hormones (48–50). Franconi et al. (48) suggested that there was a gender-specific mechanism involved in the development of diabetes complications due to the influence of sex hormones on glucose homeostasis, with women experiencing faster progression of complications and deriving less benefit from treatment compared to men. Oikonomou et al. (49) proposed that females had different risk factors and typically experienced less favorable treatment outcomes compared to males. Although we cannot explain the exact reasons for these sex differences, our results may suggest that females may require higher levels of co-exposure to multiple vitamins to achieve a greater effect. However, further research is needed to prove our speculation.

Overall, this study has several strengths, such as the prospective design and the more realistic pattern of multivitamin co-exposure generated by the unsupervised K-means clustering method. However, there are still some limitations. First, the measurement of serum vitamins was based on baseline assessments, which may not accurately reflect long-term status. Second, the long follow-up duration and potential non-compliance could lead to attrition bias or changes in known variables that may affect the outcomes. Third, the clustering results only reflect the levels of vitamins but not the categories of vitamins, and therefore the findings cannot reflect the contribution value of each vitamin. Finally, due to sample size limitations, only the relationship between co-exposure to multiple vitamins and the risk of all-cause mortality in diabetes patients was explored, but not the relationship with the mortality risk from a specific cause of death.

## 5 Conclusion

Although the individual effects of vitamins on the risk of all-cause mortality in diabetes patients vary, high levels of co-exposure to multiple vitamins can significantly reduce this risk. However, this impact may be influenced by population characteristics. Therefore, when developing intervention strategies for co-exposure to multiple vitamins in diabetes patients, it is important to consider both the individual dosages of vitamins and design the most appropriate co-exposure dosage based on population characteristics to improve the quality of life and longevity of diabetic patients.

## Data availability statement

The original contributions presented in the study are included in the article/**Supplementary Material**. Further inquiries can be directed to the corresponding authors.

## Ethics statement

The studies involving humans were approved by Ethics Committee of the Shenyang Center for Disease Control and Prevention. The studies were conducted in accordance with the local legislation and institutional requirements. The study is based on open-source data and is therefore free from ethical issues and other conflicts of interest. Research involving public data was approved by the Ethics Committee of the Shenyang Center for Disease Control and Prevention. Written informed consent for participation was not required from the participants or the participants’ legal guardians/next of kin in accordance with the national legislation and institutional requirements. Written informed consent was obtained from the individual(s) for the publication of any potentially identifiable images or data included in this article.

## Author contributions

LZ: Conceptualization, Data curation, Methodology, Validation, Visualization, Writing – original draft, Writing – review & editing. JZ: Data curation, Methodology, Software, Visualization, Writing – original draft. DZ: Data curation, Methodology, Writing – original draft. YY: Data curation, Validation, Writing – original draft. MJ: Data curation, Validation, Writing – original draft. HL: Data curation, Validation, Writing – original draft. JL: Data curation, Software, Writing – original draft. ML: Data curation, Software, Writing – original draft. ZZ: Conceptualization, Project administration, Supervision, Validation, Writing – review & editing. LG: Conceptualization, Data curation, Methodology, Project administration, Software, Supervision, Validation, Visualization, Writing – original draft, Writing – review & editing.
