# Supplementary material for: Co-exposure to multiple vitamins and the risk of all-cause mortality in patients with diabetes
Source: Front Endocrinol (Lausanne). 2023 Sep 19;14:1254133. doi: 10.3389/fendo.2023.1254133 (PMC10546318; doi:10.3389/fendo.2023.1254133)
Supplement: Supplementary file 1 [file DataSheet_1.docx]

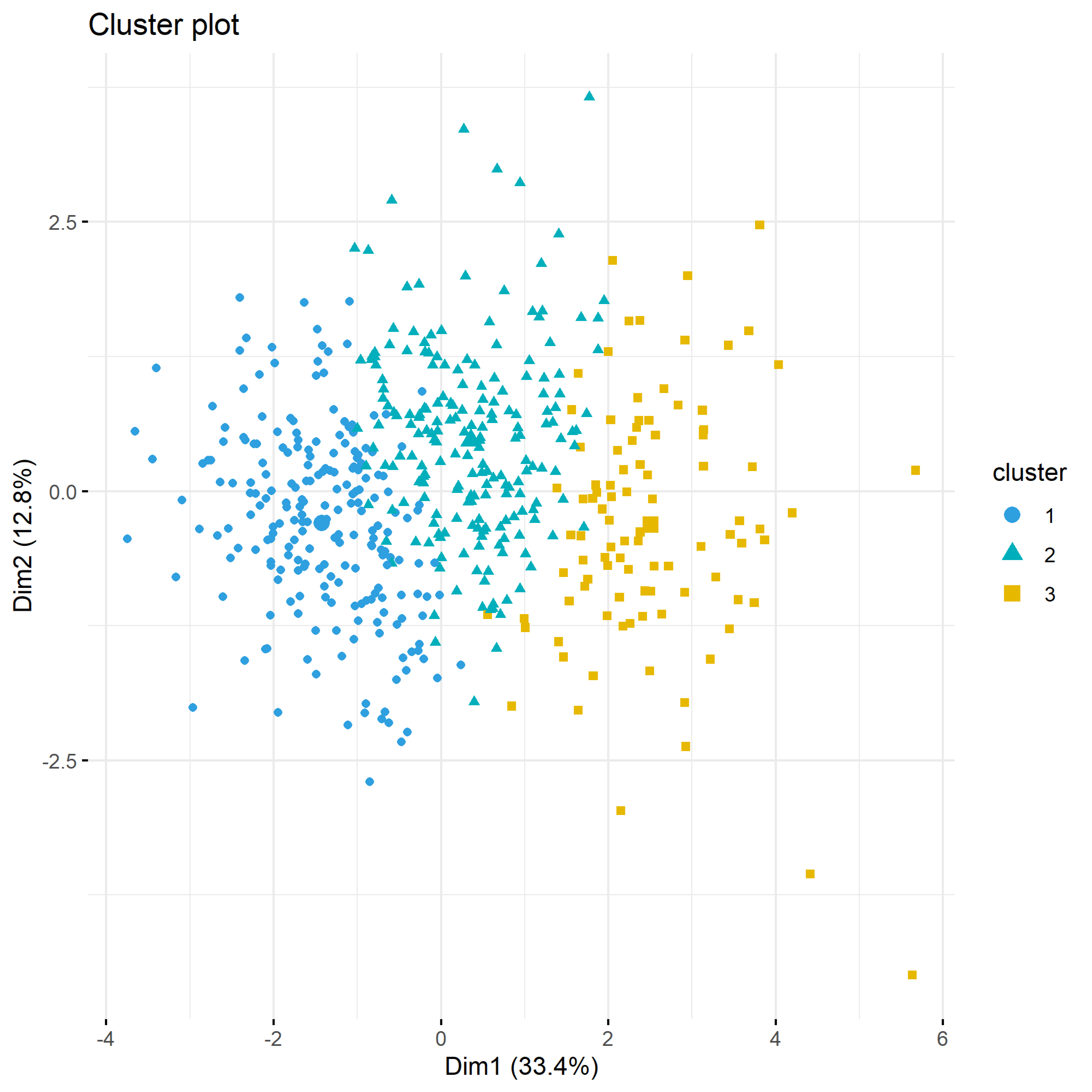


**Supplementary Figure 1** K-means clustering results of serum 8 vitamins in patients with diabetes.

Blue dots represent the first cluster (low-level group with co-exposure to multiple vitamins); green dots represent the second cluster (moderate-level group with co-exposure to multiple vitamins); yellow dots represent the third cluster (high-level group with co-exposure to multiple vitamins).

| **Supplementary Table 1** The Cox proportional hazards analysis results for the co-exposure to multiple vitamins and all-cause mortality in diabetes patients using data without weighted adjustments (sensitivity analysis) | | | | |
| --- | --- | --- | --- | --- |
| Variable | Model 1 | Model 2 | Model 3 | Model 4 |
| Cluster 1 | 1.00 | 1.00 | 1.00 | 1.00 |
| Cluster 2 | 0.81 **(**0.60, 1.09**)** | 0.71 **(**0.52, 0.97**)** | 0.83 **(**0.59, 1.16**)** | 0.82 **(**0.59, 1.16**)** |
| Cluster 3 | 0.90 **(**0.62, 1.30**)** | 0.57 (0.38, 0.85) | 0.63 (0.42, 0.95) | 0.61 **(**0.40, 0.93**)** |
| Cluster 1, low-level group with co-exposure to multiple vitamins; Cluster 2, moderate-level group with co-exposure to multiple vitamins; Cluster 3, high-level group with co-exposure to multiple vitamins.  Model 1, unadjusted for covariates; Model 2, adjusted for age, gender, education level, and PIR; Model3, further adjusted for physical activity, smoking, alcohol consumption, BMI, and HEI-2015; Model4, further adjusted for hypertension, high cholesterol, liver disease, and kidney disease. | | | | |

| **Supplementary Table 2** The Cox proportional hazards analysis results for the co-exposure to multiple vitamins and all-cause mortality in diabetes patients using death data until December 31, 2015 (sensitivity analysis) | | | | |
| --- | --- | --- | --- | --- |
| Variable | Model 1 | Model 2 | Model 3 | Model 4 |
| Cluster 1 | 1.00 | 1.00 | 1.00 | 1.00 |
| Cluster 2 | 1.02 **(**0.64, 1.62**)** | 0.80 **(**0.51, 1.25**)** | 0.98 **(**0.62, 1.53**)** | 0.93(0.64, 1.35) |
| Cluster 3 | 0.88 **(**0.50, 1.55**)** | 0.50 (0.28, 0.88) | 0.53 (0.28, 1.00) | 0.52 **(**0.27, 1.00**)** |
| Cluster 1, low-level group with co-exposure to multiple vitamins; Cluster 2, moderate-level group with co-exposure to multiple vitamins; Cluster 3, high-level group with co-exposure to multiple vitamins.  Model 1, unadjusted for covariates; Model 2, adjusted for age, gender, education level, and PIR; Model3, further adjusted for physical activity, smoking, alcohol consumption, BMI, and HEI-2015; Model4, further adjusted for hypertension, high cholesterol, liver disease, and kidney disease. | | | | |
